# The causal relationship of type 1 diabetes and its complications on ingrown nails: Insights from a multivariable Mendelian randomization study

**DOI:** 10.1097/MD.0000000000041719

**Published:** 2025-03-14

**Authors:** Simin He, Siliang Xue, Wei Chen, Zhipeng Deng, Erlong Li, Jianbo Zhao

**Affiliations:** a Department of Dermatology, West China Hospital, Sichuan University, Chengdu, China; b Laboratory of Dermatology, Clinical Institute of Inflammation and Immunology, Frontiers Science Center for Disease-related Molecular Network, West China Hospital, Sichuan University, Chengdu, China; c College of Information and Communication Engineering, Harbin Engineering University, Chengdu, Sichuan, China.

**Keywords:** diabetes, ingrown nails, multi-variable Mendelian randomization study, type 1 diabetes, type 1 diabetes with complications, type 2 diabetes

## Abstract

Diabetic patients often experience ingrown nails. Nevertheless, the causal relationship between different types of diabetes, their complications, and the occurrence of ingrown nails has yet to be fully clarified. We utilized single nucleotide polymorphisms as instrumental variables for exposure and performed Mendelian randomization (MR) analysis to ascertain the causal relationship between different types of diabetes and ingrown nails. Databases of diabetes were represented through 3 categories: type 1 diabetes, type 1 diabetes with or without complications, and type 2 diabetes, encompassing a cohort of 1,575,134 individuals of European descent. Following our analysis of the MR results, we determined the overall effect size and causal linkage related to type 1 diabetes and its complications. Type 1 diabetes had been shown to increase the risk of ingrown nails with an odds ratio of 1.09 (95% confidence interval: 1.05–1.12; *P* < .001). The MR results demonstrated a causal relationship between type 1 diabetes with or without complications and ingrown nails, revealing distinct odds ratios. In contrast, the MR findings indicated an absence of a specific causal relationship between type 2 diabetes and ingrown nails. All our sensitivity analyses have proven the validity and reliability of the results. This study indicates that individuals with type 1 diabetes are more likely to develop an ingrown nail compared to those without. Compared to patients without complications of type 1 diabetes, those who have complications are more likely to get an ingrown nail. Meanwhile, our current data do not support a specific causal relationship between type 2 diabetes and ingrown nails.

## 1. Introduction

An ingrown nail is a prevalent medical condition characterized by inflammation of the tissue along the edge of the nail, often leading to significant pain and difficulty in walking.^[1,2]^ This condition may affect quality of life and have serious social implications. When the lateral nail fold is penetrated by the edge of the nail plate, the damaged skin attempts to heal itself by producing highly vascular granulation tissue, which protrudes beyond the nail plate.^[3,4]^ Ingrown nails are classified into 4 stages: mild erythema, edema, and pain; prominent erythema, edema, and pain, accompanied by discharge; erythema, edema, pain, and discharge, along with skin hypertrophy and granulation tissue formation in the area of the lateral skin fold; and serious chronic deformity of the toenail.^[5]^ Conservative approaches include appropriate nail cutting, packing of the nail sulcus, and nail bracing, which are recommended for managing mild-to-moderate ingrown nails.^[6,7]^ When conservative treatment fails or in cases of nail deformity or severe infections, a surgical approach is often recommended.

Therefore, foot lesions warrant special attention in the diabetic population. Previous studies have identified diabetes as a factor in the etiology of ingrown nails, which are commonly observed in diabetic patients and cited as one of the risk factors for developing foot lesions.^[8]^ The primary etiological factors of diabetic foot complications are as follows: sensorimotor polyneuropathy, peripheral vascular disease, tissue trauma and breakdown, immune dysfunction, and infections.^[9]^ Previous studies have shown a high prevalence of foot abnormalities in adolescents with type 1 diabetes (T1D). Specifically, 18% of patients exhibit a decreased range of motion in the first metatarsophalangeal joint, which is associated with microvascular lesions.^[10]^ The neurological disability score also indicated that 17% of patients with T1D (median age: 23 years) had peripheral neuropathy.^[11]^ Recently, a report noted that despite a shorter duration of diabetes and lower HbA1c levels, youths with type 2 diabetes (T2D) had similar rates of peripheral and autonomic neuropathy to those with T1D.^[12]^ In individuals with diabetes, the loss of protective sensation may cause a delay in recognizing an ingrown nail. This delay can potentially make the ingrown nail less obvious and increase the morbidity.^[13]^ Furthermore, peripheral vascular disease in patients with diabetes has been shown to increase nail thickness and disrupt nail structure, making these patients more susceptible to bacterial and fungal infections of the feet, which may accelerate the progression of ingrown nails. To date, no study has explored the different types of diabetes and their causal relationships with ingrown nails.

Conventional observational studies are prone to confounding factors and reverse causation biases. To address these limitations, Mendelian randomization (MR) uses genetic variants as instrumental variables (IVs) to infer causal relationships.^[14,15]^ Using MR not only overcomes the limitations of observational studies by mimicking a randomized controlled trial but also offers evidence beyond clinical studies to establish a causal association between diabetes and ingrown nails. Given the distinct natural histories of T1D and T2D, previous observational evidence is insufficient and cannot be directly interpreted as causal owing to potential uncontrolled confounding and reverse causality. In this study, we conducted a multivariable MR. Our aim was to investigate the independent causal effects. These effects were of different types of diabetes and their complications on ingrown nails.

## 2. Materials and methods

### 2.1. Study design

**Figure 1.**
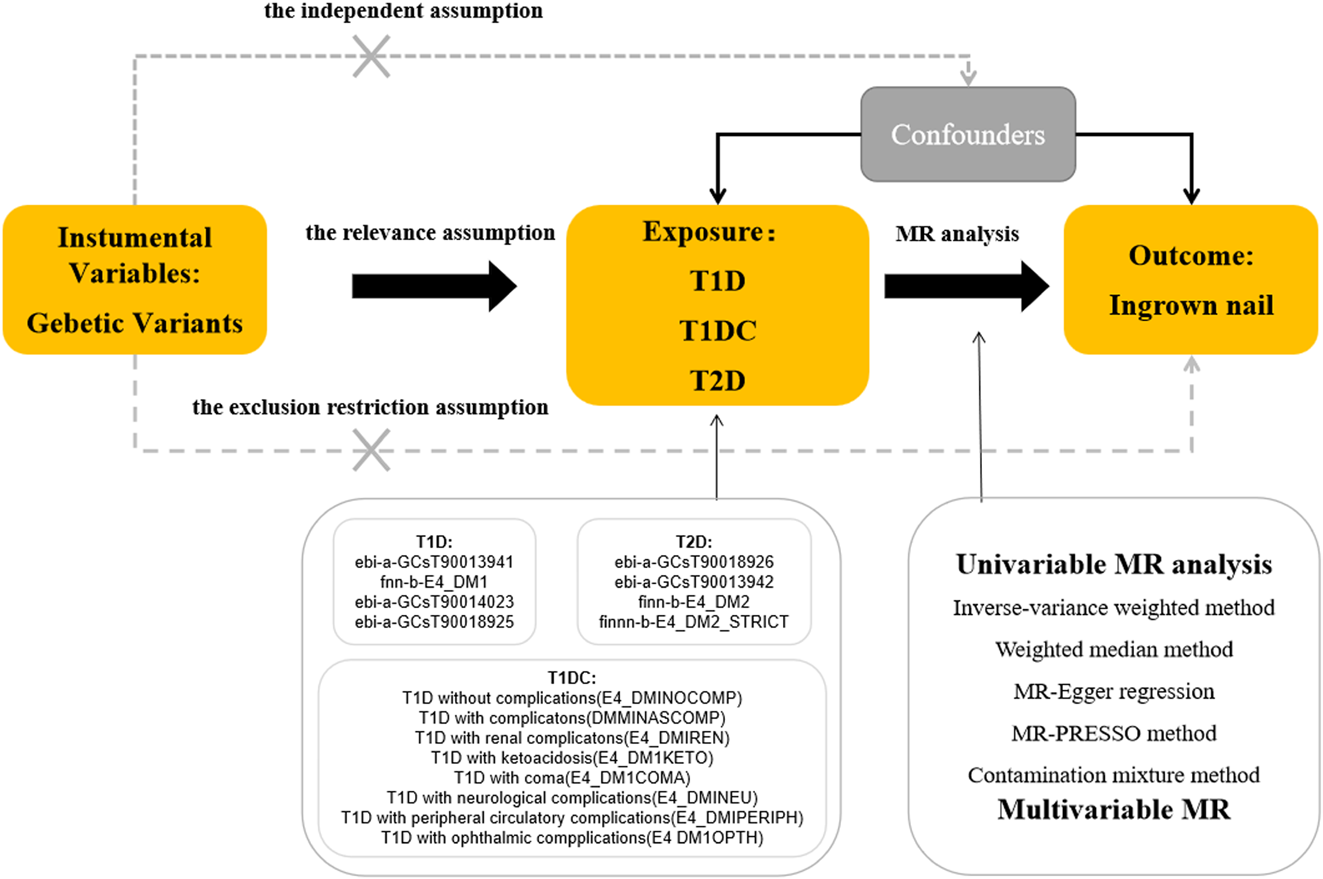
A brief description of this Mendelian randomization (MR) design. T1D = type 1 diabetes, T1DC = type 1 diabetes with complication, T2D = type 2 diabetes.

First, the 2-sample MR method was employed to investigate the causal relationship between T1D and T2D and the risk of ingrown nails. In the second part, a meta-analysis was conducted to calculate the weights and total effects of the different exposure datasets. In the third part, the causal relationship between 8 datasets containing diabetes with or without complications and ingrown nails was explored using the 2-sample MR approach. R software (v.4.4.0) was used, along with the *Two-Sample MR* package, the *metafor* package, and the *forestplter* package. A brief description of the MR design is shown in Figure 1.

The analytic process adhered to the Strengthening the Reporting of Observational Studies in Epidemiology-MR guidelines,^[16]^ which rely on 3 hypotheses of MR^[17]^: the correlation hypothesis, which suggests that exposure is closely related to heredity; the independence hypothesis, where genetic variation is independent of confounding factors; and the exclusivity hypothesis, which suggests that genetic variation affects results only through exposure.

### 2.2. Selection of IVs and data source

T1D data were obtained from 4 datasets derived from the Integrative Epidemiology Unit Open genome-wide association studies database as detailed in Table 1; therefore, no additional ethical review was required. All participants were of European descent; thus, racial bias was excluded. We selected the corresponding data following the steps outlined in Figure 1 and created IVs representing exposure effects using genetic sites associated with mRNA expression. Single nucleotide polymorphism (SNP) from the exposed data were clustered using the parameter *P* = 5e-8, which ensured the validity of the first assumption of MR. *R*^2^ = 0.001 and a window length of 10 Mb based on the linkage disequilibrium hypothesis,^[18]^ which ensured the validity of the second assumption of MR. The type 1 diabetes with complication (T1DC) was obtained from the Finnish database, as shown in Table 1. The same screening criteria as those used for T1D were applied to the exposed data. Four datasets for T2D were obtained from the Integrative Epidemiology Unit Open genome-wide association studies database, as detailed in Table 1. The subjects were males and females of European descent, encompassing a total of 1,575,134 individuals and 111,597,523 SNPs. The same screening criteria used for T1D were also applied to the exposed T2D data. Ingrown nail data were obtained from the NA consortium. This dataset included males and females of European descent, with 16,380,452 SNPs, providing relevant data on ingrown nails.

**Table 1:** Information on data included in the study.

Phenotypes/ID	Data source	Study information/PMID	Cases/controls	Author/yr
T1D: ebi-a-GCST90013941	UKB, Finnish database	European/34,017,140	407,746	Mbatchou J/2021
T1D: finn-b-E4_DM1	Finnish database	European/34012112	5928/183,185	NA/2021
T1D: ebi-a-GCST90014023	UKB, Finnish database	European	18,942	Chiou J/2021
T1D: ebi-a-GCST90018925	UKB, Finnish database	European/34,594,039	6447/451,248	NA/2022
T1D with complications: DM1NASCOMP	Finnish database	European	6234/308,280	NA/2022
T1D without complications: E4_DM1NOCOMP	Finnish database	European	4918/183,185	NA/2021
T1D with renal complications: E4_DM1REN	Finnish database	European	1579/308,280	NA/2022
T1D with ketoacidosis: E4_DM1KETO	Finnish database	European	2102/308,280	NA/2022
T1D with coma: E4_DM1COMA	Finnish database	European	2050/308,280	NA/2022
T1D with neurological complications: E4_DM1NEU	Finnish database	European	1077/308,280	NA/2022
T1D with peripheral circulatory complications: E4_DM1PERIPH	Finnish database	European	669/308,280	NA/2022
T1D with ophthalmic complications: E4_DM1OPTH	Finnish database	European	5202/308,280	NA/2022
T2D: ebi-a-GCST90018926	Finnish database	European/34,594,039	38,841/451,248	NA/2021
T2D: ebi-a-GCST90013942	Finnish database	European/34017140	406,831	NA/2021
T2D: finn-b-E4_DM2	Finnish database	European	32,469/183,185	NA/2021
T2D: finn-b-E4_DM2_STRICT	Finnish database	European	29,166/183,185	NA/2021
Ingrown nail: finn-b-L12_NAIL_INGROW	Finnish database	European	762/211,139	NA/2021

ID = identity, NA = not available, PMID = PubMed identity, T1D = type 1 diabetes, T2D = type 2 diabetes, UKB = United Kingdom Biobank.

### 2.3. MR analysis

Outcome and exposure data were matched, repeated palindromic SNPs were removed, and the remaining SNPs were retained as instruments. The statistical power of all SNPs was assessed using the F statistic,^[19,20]^ and those >10 were considered strong IVs. The F is calculated as follows:


$$\begin{array}{l} F=\frac{(n-k-1)kR^{2}}{1-R^{2}} \end{array}$$

While n was the sample size, k was 1, and the coefficient of determination $R^{2}$ served as a metric to measure the proportion of variation explained by individual SNPs. The $R^{2}$ was calculated using the following formula:


$$\begin{array}{l} R^{2}=\frac{2\beta^{2}\cdot EAF\cdot (1-EAF)}{2\beta^{2}\cdot EAF\cdot (1-EAF)+2nSE^{2}\cdot EAF\cdot (1-EAF)} \end{array}$$

Where β was the Effect size, SE is the standard deviation, and EAF is the total of 5 MR Methods used in the MR Process: random-effects inverse-variance weighted (IVW), MR-Egger, weighted median, simple mode, and weighted mode.

We use the IVW method to estimate the overall effect size. This method calculates the causal effect of exposure on the outcome. It does this by weighted averaging the effect sizes obtained from IV estimates.^[21]^ When there is no directional pleiotropy and heterogeneity between exposure and outcome, estimates from this method are reported to be reasonably accurate. We investigated the potential presence of horizontal pleiotropy among the IVs using the MR-Egger regression method and MR-PRESSO. Evidence of horizontal pleiotropy was determined by a significant deviation of the MR-Egger intercept from zero, with a *P*_1_ value of ≤ .05. The weighted median method yields an unbiased causal estimate provided that at least 50% of the genetic instruments satisfy the 3 assumptions of MR.^[22]^ The simple mode method clusters the causal effect estimates of individual SNPs and selects the largest SNP cluster to estimate the causal effect. In contrast, the weighted-mode method applies weights to each SNP during the clustering process.^[23]^ Forest plots visually displayed effect sizes and statistical significance, whereas scatter plots illustrated causal direction. A causal relationship between the factor and outcome was indicated when the confidence interval of the IVW method did not cross the null line. The *leave-one-out* method was used to assess the impact of each SNP on the overall causal estimate; if a single SNP significantly affected the estimate, it was removed for reevaluation. Funnel plots were used to determine potential bias.

### 2.4. Meta-analysis

We conducted a meta-analysis on T1D outcomes from various datasets. The purpose was to generate a consolidated and more stable effect.^[24]^ The aggregated effect size comprehensively estimated the combined effect of all IVs. It described the causal impact of T1D on ingrown nails. Since ingrown nail was a binary variable, we expressed the meta-analysis effect using odds ratios (ORs). The OR quantified the influence of T1D incidence on the risk of ingrown nail disease; an OR of 1 indicated no impact, values below 1 suggested a detrimental effect, and those above 1 indicated a positive correlation. The results were evaluated for significance using a *P* threshold of 0.05, and outcomes were considered significant if *P* < .05. Heterogeneity was assessed using *I*²; values below 50% suggested homogeneity and favored a fixed-effects model, while values exceeding 50% indicated heterogeneity and recommended a random-effects model.

## 3. Results

### 3.1. The effect of T1D on the risk of ingrown nails

**Figure 2.**
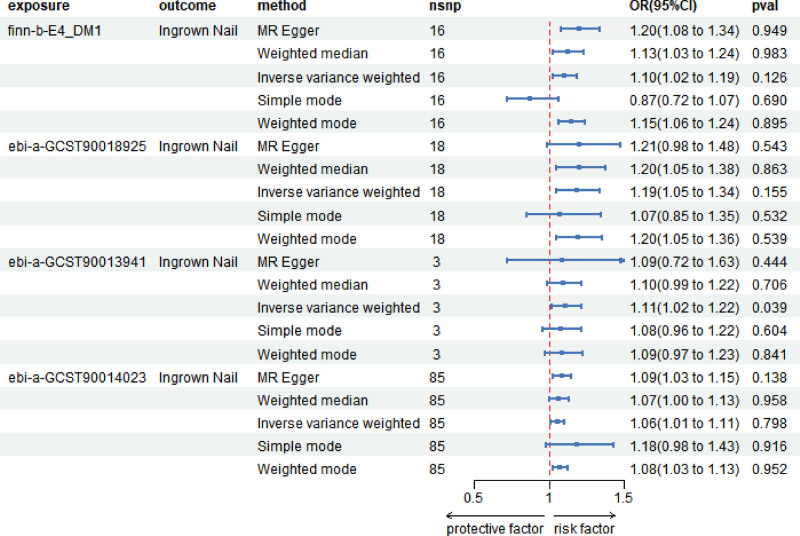
Forest plot depicting the Mendelian randomization (MR) results of the type 1 diabetes (T1D) datasets across various methodologies. The first column shows the IDs of T1D from different datasets. CI = confidence interval, OR = odds ratio.

After conducting 2 MR analyses, all SNPs exhibited F-statistics >10, confirming their strength as instruments. The ORs between T1D datasets and ingrown nails were estimated as follows: 1.10 (95% confidence interval [CI]: 1.02–1.19, *P* = .010), 1.19 (95% CI: 1.05–1.34, *P* = .006), 1.11 (95% CI: 1.02–1.22, *P* = .022), and 1.06 (95% CI: 1.01–1.11, *P* = .015). Both individual dataset efficacy and aggregated effect consistently demonstrated a positive association between T1D and the risk of ingrown nails. Figure 2 presents a forest plot of the MR results across various datasets. The confidence interval from the IVW method did not intersect the null line, indicating a significant causal relationship between T1D and ingrown nails. The regression line and *P*_1_ > .05 revealed no notable horizontal pleiotropy (see Figure S1, Supplemental Digital Content, http://links.lww.com/MD/O479, which visually demonstrates the existence of a causal relationship and proves the validity of the IVs as the scatter plot of T1D). The leave-one-out method confirms the robustness of the study (see Figure S2, Supplemental Digital Content, http://links.lww.com/MD/O479, which confirms that no single SNP significantly influenced the results). The funnel plot indicates no evidence of bias (see Figure S3, Supplemental Digital Content, http://links.lww.com/MD/O479, in which the points basically assume a funnel shape and are symmetrical).

**Figure 3.**
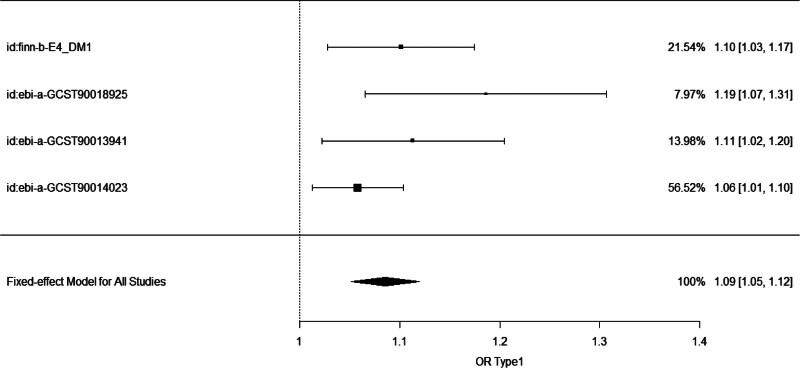
Forest plot of type 1 diabetes (T1D) odds ratios (ORs) by inverse-variance weighted from different datasets and combined OR. The first column shows the IDs of T1D from different datasets.

MR data from the 4 datasets were used for the meta-analysis. The overall OR between T1D and ingrown nails was estimated at 1.09 (95% CI: 1.05–1.12, *P* < .001), as shown in Figure 3. The *I*² value was 28%, and a fixed-effects model was used with a significance level of *P* < .001. The data indicated low heterogeneity in the meta-analysis, and the overall effect was statistically significant.

### 3.2. The effect of T1DC on the risk of ingrown nails

**Figure 4.**
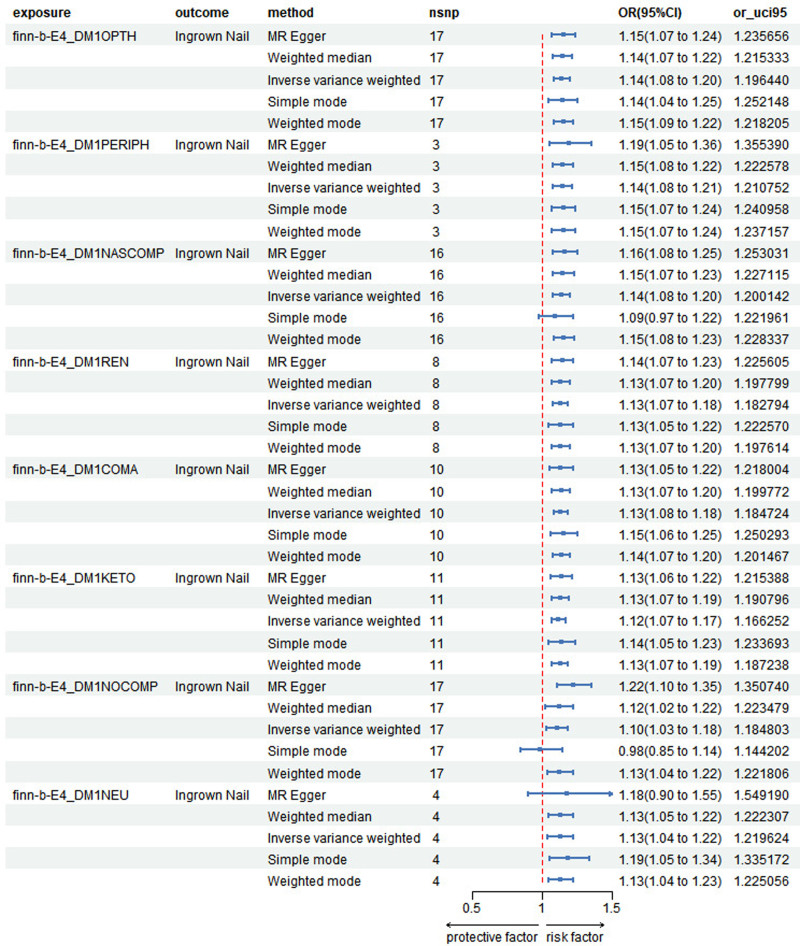
Forest plot of the Mendelian randomization (MR) results between type 1 diabetes (T1D) complications and ingrown nails. The first column shows the IDs of T1D with/without complications from different datasets. CI = confidence interval.

All SNPs had an F-statistics >10, indicating that they were all strong instruments. The MR results illustrated the relationship between the 8 T1DCs and ingrown nails, showing distinct ORs for different complications, as depicted in Figure 4. It was evident that all T1DCs were positively correlated with the risk of ingrown nails, which was statistically significant based on *P* values. The ORs were 1.14 (95% CI: 1.08–1.20, *P* < .001) for ophthalmic complications, 1.14 (95% CI: 1.08–1.21, *P* < .001) for peripheral circulatory complications, 1.14 (95% CI: 1.08–1.20, *P* < .001) for other specified/multiple/unspecified complications, and 1.13 (95% CI: 1.07–1.18, *P* < .001) for renal complications.

The OR of T1D with complications was estimated at 1.13 (95% CI: 1.08–1.18, *P* < .001). The OR for T1D with ketoacidosis was estimated at 1.12 (95% CI: 1.07–1.17, *P* < .001). The OR of T1D without complications was estimated at 1.10 (95% CI: 1.03–1.18, *P* = .006). The OR of T1D with neurological complications was estimated at 1.13 (95% CI: 1.04–1.22, *P* < .001). The regression line and *P*_1_ > .05 demonstrated no specific horizontal pleiotropy (see Figure S4, Supplemental Digital Content, http://links.lww.com/MD/O479, which visually demonstrates the existence of a causal relationship and proves the validity of the IVs as the scatter plot of T1DCs). The leave-one-out analysis proves the robustness of the study (see Figure S5, Supplemental Digital Content, http://links.lww.com/MD/O479, which confirms that no single SNP significantly influenced the results). The funnel plot indicated no specific bias (see Figure S6, Supplemental Digital Content, http://links.lww.com/MD/O479, in which the points basically assume a funnel shape and are symmetrical).

### 3.3. The effect of T2D on the risk of ingrown nails

**Figure 5.**
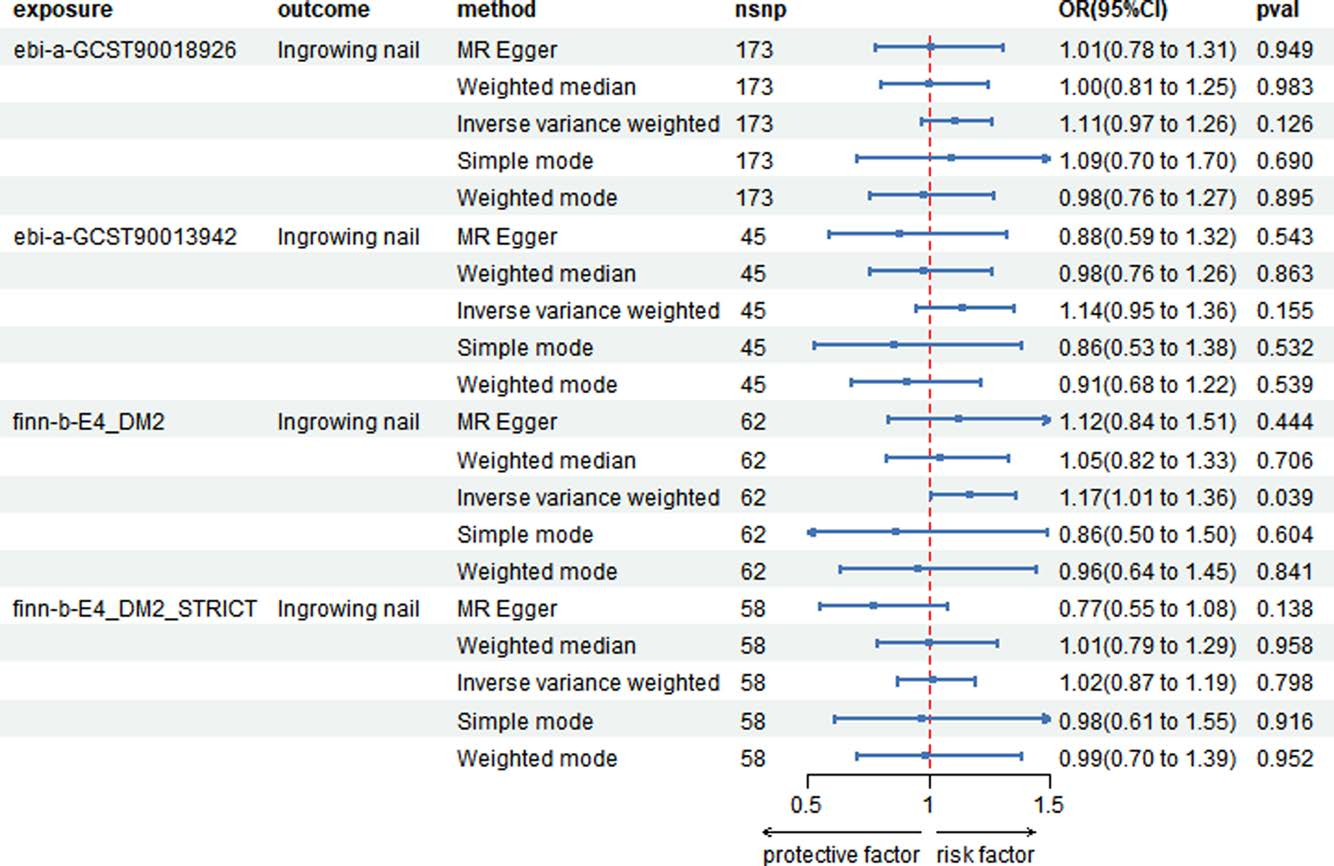
Forest plot depicting the Mendelian randomization (MR) results of the type 2 diabetes (T2D) datasets across various methodologies. The first column shows the IDs of T2D from different datasets. CI = confidence interval, OR = odds ratio.

The MVMR results indicated that T2D was not a potential influencing factor for ingrown nails. The ORs between the T2D datasets and the ingrown nails are shown in Figure 5. Among the 2 T2D datasets, the *P* values for 1 dataset’s MR results were >.05, indicating no statistical significance. However, for the finn_b_E4_DM2 dataset, the estimated OR was 1.17 (95% CI: 1.01–1.36, *P* < .001), showing a different result. However, the final dataset, finn_b_E4_DM2_STRICT, which is a strictly defined dataset for T2D, did not show a specific causal relationship with ingrown nails. The analysis concluded that there is no specific causal relationship between T2D and ingrown nails.

## 4. Discussion

In foot examinations of diabetic patients, the prevalence of ingrown nails has been reported to range from 13% to 32% in several studies, whereas in the general population, it ranges from 2.5% to 5%.^[25–28]^ The duration of diabetes among patients with ingrown nails was longer compared to those without ingrown nails (10.41 vs 9.36 years).^[13]^ Previous studies have suggested that male patients with ingrown nails with tall stature, pseudoacromegaly, or hyperhidrosis should be examined for latent diabetes.^[29]^

In this study, we used MR combined with meta-analysis to confirm the causal relationship between T1D and ingrown nails. The OR was 1.09 (95% CI: 1.05–1.12, *P* < .001). This shows that patients with T1D have a 9% higher risk of developing ingrown nails compared to those without T1D. Moreover, this difference is statistically significant. The causal relationship between T1DC and the risk of ingrown nails was also confirmed. The T1DC datasets included: T1D with complications (DMMINASCOMP), T1D without complications (E4_DMINOCOMP), T1D with renal complications (E4_DMIREN), T1D with ketoacidosis (E4_DM1KETO), T1D with coma (E4_DM1COMA), T1D with neurological complications (E4_DMINEU), T1D with peripheral circulatory complications (E4_DMIPERIPH), and T1D with ophthalmic complications (E4_DM1OPTH). These results indicated that the influence of T1D and T1DC on the risk of ingrown nails cannot be overlooked. Regarding T2D, while 1 dataset with a broad definition demonstrated a causal relationship between T2D and ingrown nails, the other 3 datasets showed no specific relationship. Analysis of MR results from various T2D datasets leads to the conclusion that the current data do not support a specific causal relationship between T2D and ingrown nails.

Diabetic complications lead to a significant disease burden. These complications include blindness, kidney dysfunction, damage to small and large blood vessels, ketoacidosis, neuropathy, and a decline in the quality of life. Microvascular complications include diabetic neuropathy (DN), retinopathy, and nephropathy. In contrast, macrovascular complications contribute to the development of cardiovascular diseases such as coronary artery disease, cerebrovascular disease, and peripheral arterial disease. Hypertension is twice as common in diabetic patients as in the general population. Diabetic patients with concomitant hypertension have a higher frequency of ingrown nails than those without.^[13,30,31]^ Micro- and macrovascular complications may impair nail unit nutrition, thereby increasing the risk of ingrown nails.^[32,33]^

DN is the most common chronic complication of diabetes, and distal symmetrical polyneuropathy is the most prevalent manifestation. Neurovascular changes, including thickening of the basement membrane, degeneration of pericytes, endothelial cell hyperplasia, and arterio-venous shunt formation, result in ischemia of the peripheral neurons.^[34]^ This ischemia then induces neuronal injury through increased reactive oxygen species.^[35]^ A previous study found that the legacy effect of glycemic control was sustained for DN,^[36]^ and a systematic review of 17 randomized control studies indicated that intensive glycemic control prevented the development of DN in patients with T1D.^[37]^ Conversely, in patients with T2D, although nerve conduction may specifically improve, intensive glycemic control does not reduce the incidence of neuropathy. Increased forefoot pressure in individuals with diabetes, particularly those with diagnosed neuropathy, can lead to subtle abnormal gait patterns and increased stress on the forefoot and toes.^[9]^ These pressures result in increased direct mechanical forces and the shearing of the nail unit against the soft tissue of the nail groove. Patients with sensory neuropathy are unable to respond promptly to pain, leading to more severe cases of ingrown nails.

Diabetic ketoacidosis and its association with coma are the most common acute hyperglycemic emergencies in patients with diabetes and are typically linked with T1D.^[38,39]^ Although diabetic ketoacidosis can also occur in T2D, our study suggests a causal association between ingrown nails and ketoacidosis and coma, specifically in T1D rather than in T2D. The mechanisms linking diabetic ketoacidosis with ingrown nails are not well understood. To date, no study has focused on this relationship, making our study the first to report such an association.

To the best of our knowledge, this is the first study to investigate the causal association between T1D/T1DC and ingrown nails. This study addresses a gap in the current human-level research on the causal relationship between T1D/T1DC and ingrown nails. The use of the MVMR method helped mitigate confounding biases and provided robust causal effect estimates.

The strengths of this study include the following: rigorous examination of the causal relationship between diabetes and ingrown nails using the MR method. Evidence suggests that T1D with complications has a greater impact on ingrown nails than T1D without complications, although further evidence is required to confirm this. Simultaneously, we conducted an analysis of T2D and found no clear causal relationship with the embedded compounds, ruling out certain hypotheses. Contribution to the clinical field by offering new insights into the epidemiological basis of the relationship between diabetes and ingrown nails.

However, this study also has several limitations: the use of fixed screening parameters might result in systematic errors and residual confounding. The causal relationship between T1D and ingrown nails is highly specific, whereas the causal relationship between T2D and ingrown nails has not been specifically proven. The internal relationship between these needs to be further studied. There are limited clinical reports and studies on the association between diabetes and ingrown nails, indicating the need for further data to supplement and validate this research.

## 5. Conclusion

This study indicates that T1D is associated with an increased risk of nail embedding, and the incidence of ingrown nails is 9% higher in patients with T1D than in those without. Patients with T1DC are more likely to develop ingrown nails than those with T1D, but there are no complications. The current data does not support a specific causal relationship between T2D and ingrown nails.

## Author contributions

**Conceptualization:** Simin He, Siliang Xue.

**Data curation:** Simin He, Wei Chen, Erlong Li.

**Formal analysis:** Simin He, Zhipeng Deng.

**Methodology:** Simin He, Wei Chen, Jianbo Zhao.

**Software:** Simin He, Wei Chen, Jianbo Zhao.

**Visualization:** Simin He, Erlong Li, Jianbo Zhao.

**Writing – original draft:** Simin He.

**Writing – review & editing:** Simin He, Siliang Xue, Jianbo Zhao.

**Funding acquisition:** Siliang Xue, Jianbo Zhao.

**Project administration:** Siliang Xue.

**Resources:** Siliang Xue, Zhipeng Deng, Erlong Li.

**Supervision:** Siliang Xue.

**Investigation:** Zhipeng Deng.

## Supplementary Material


