# Supplementary material for: The causal relationship of type 1 diabetes and its complications on ingrown nails: Insights from a multivariable Mendelian randomization study
Source: Medicine (Baltimore). 2025 Mar 14;104(11):e41719. doi: 10.1097/MD.0000000000041719 (PMC11922480; doi:10.1097/MD.0000000000041719)
Supplement: Supplementary file 1 [file medi-104-e41719-s001.docx]

**Supplemental materials**


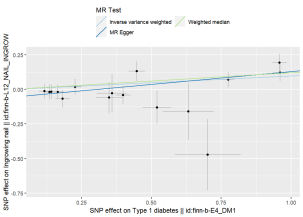

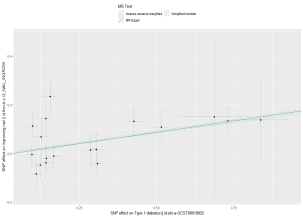

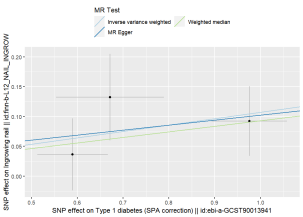

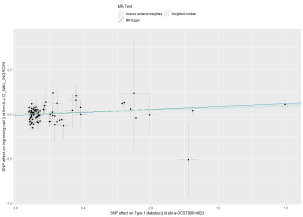


(a) (b) (c) (d)

S.1 Scatter plots of T1D: (a)Scatter plots of finn-b-E4_DM1, (b)Scatter plots of ebi-a-GCST90018925, (c)Scatter plots of ebi-a-GCST90013941, (d)Scatter plots of ebi-a-GCST90014023.

**Supplemental materials**


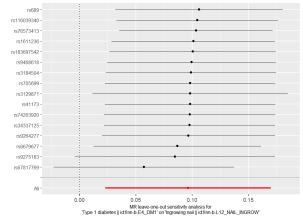

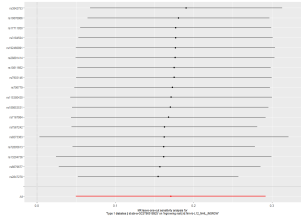

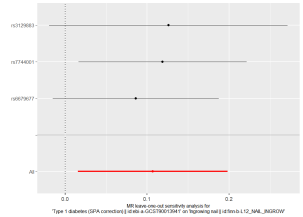

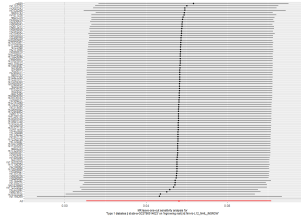


S.2 Leave-one-out plots of T1D from different datasets: (a)Leave-one-out plot of finn-b-E4_DM1, (b)Leave-one-out plot of ebi-a-GCST90018925, (c)Leave-one-out plot of ebi-a-GCST90013941, (d)Leave-one-out plot of ebi-a-GCST90014023

**Supplemental materials**


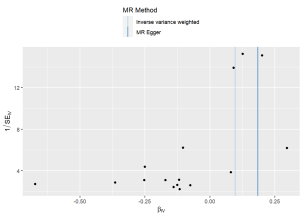

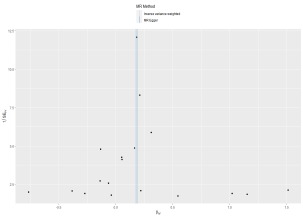

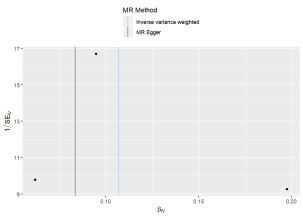

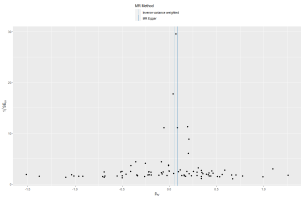


(a) (b) (c) (d)

S.3 Funnel plots of T1D from different datasets: (a)Funnel plot of finn-b-E4_DM1, (b)Funnel plot of ebi-a-GCST90018925, (c)Funnel plot of ebi-a-GCST90013941, (d)Funnel plot of ebi-a-GCST90014023

**Supplemental materials**


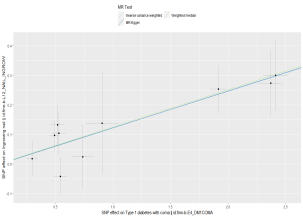

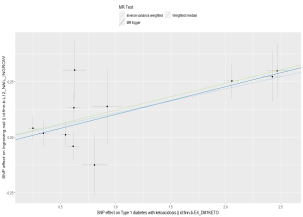

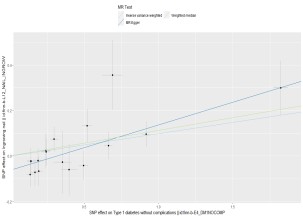

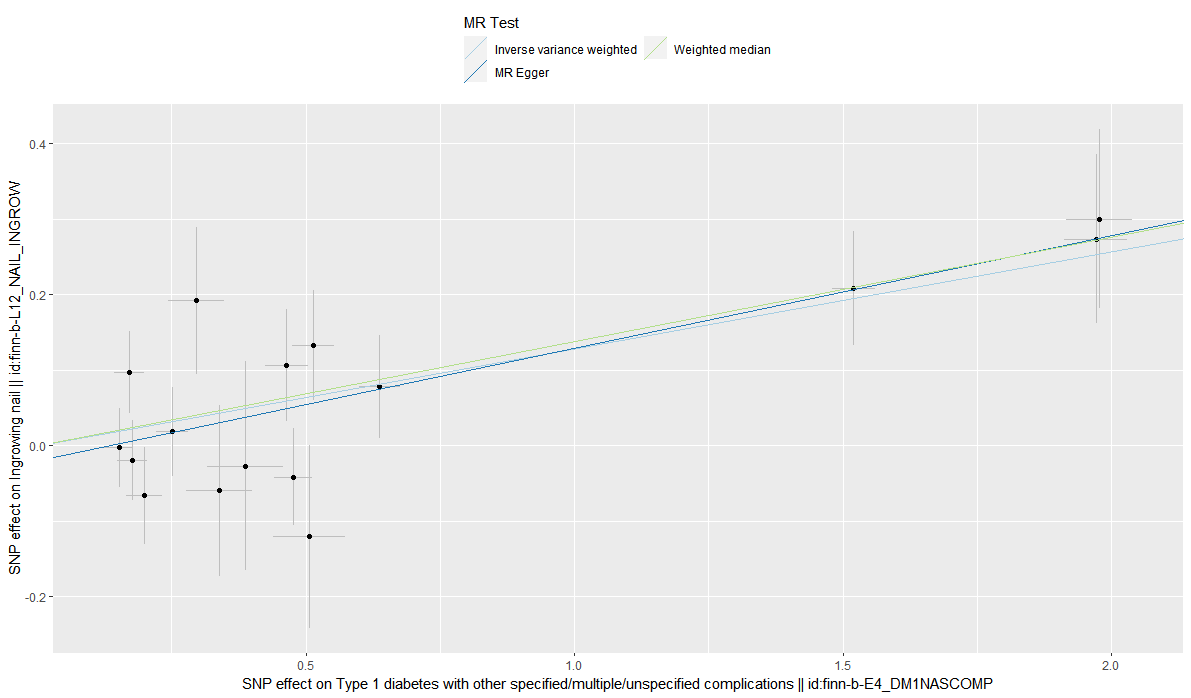


(e) (f) (g) (h)

S.4 Scatter plots of T1DCs: (a)Scatter plot of DM1OPTH, (b)Scatter plot of DM1PERIPH, (c)Scatter plot of DM1NASCOMP, (d)Scatter plot of DM1REN, (e)Scatter plot of DMICOMA, (f)Scatter plot of DM1KETO, (g)Scatter plot of DM1NOCOMP, (h)Scatter plot of DM1NEU

**Supplemental materials**


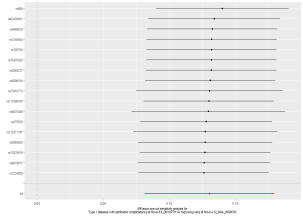

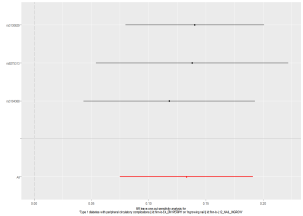

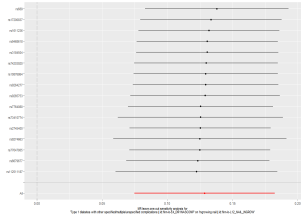

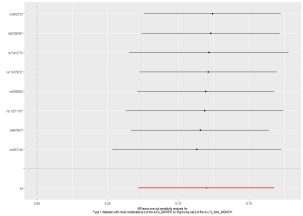


(a) (b) (c) (d)


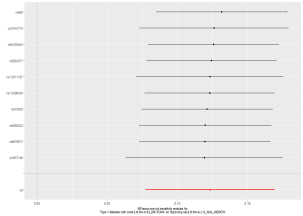

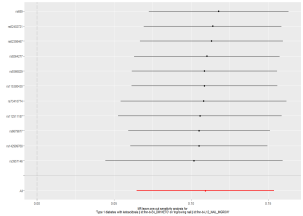

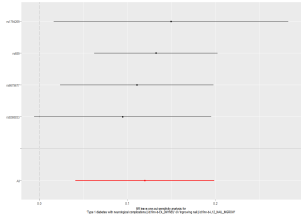

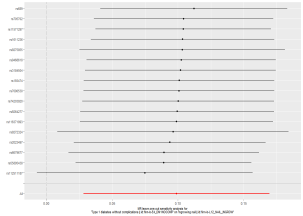


(e) (f) (g) (h)

S.5 Leave-one-out plots of T1DCs from different datasets: (a)Leave-one-out plot of DM1OPTH, (b)Leave-one-out plot of DM1PERIPH, (c)Leave-one-out plot of DM1NASCOMP, (d)Leave-one-out plot of DM1REN, (e)Leave-one-out plot of DMICOMA, (f)Leave-one-out plot of DM1KETO, (g)Leave-one-out plot of DM1NOCOMP, (h)Leave-one-out plot of DM1NEU

**Supplemental materials**


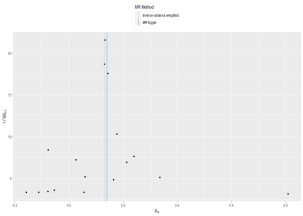

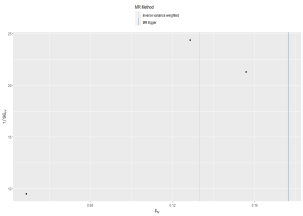

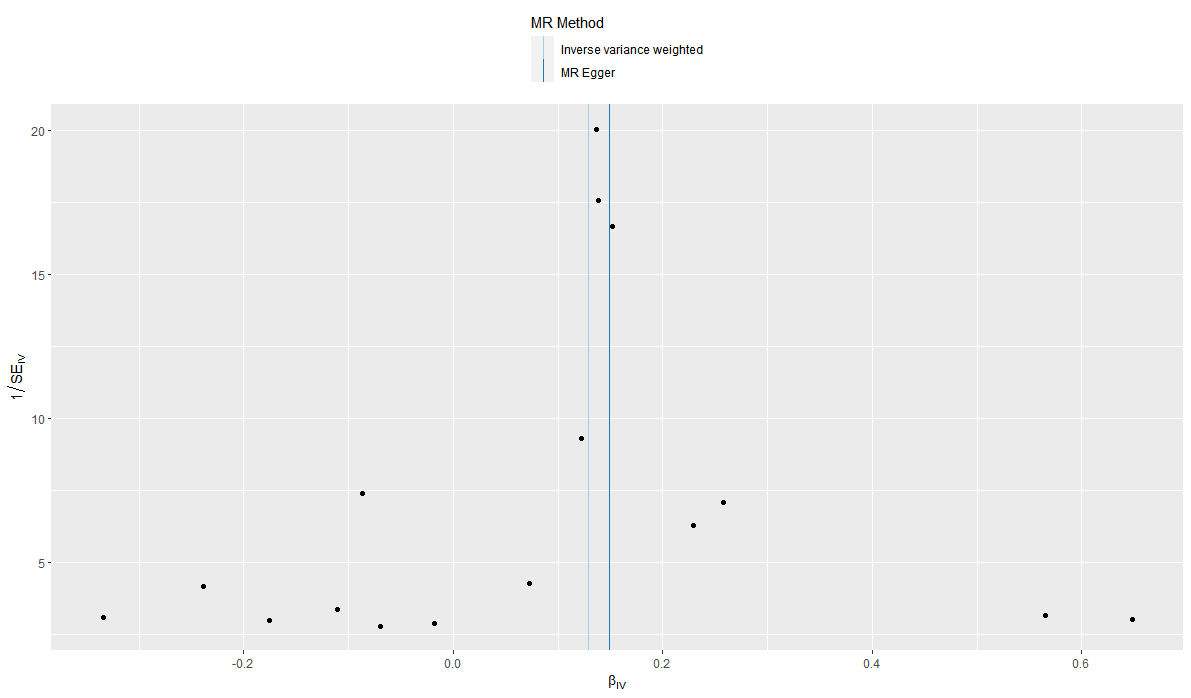

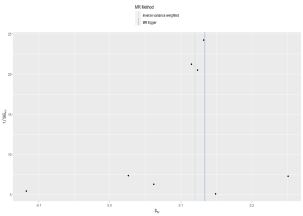


(a) (b) (c) (d)


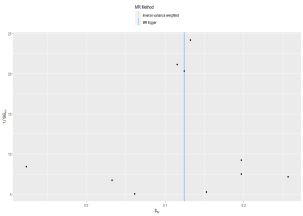

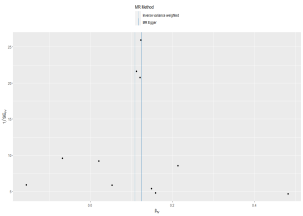

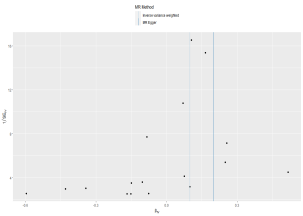

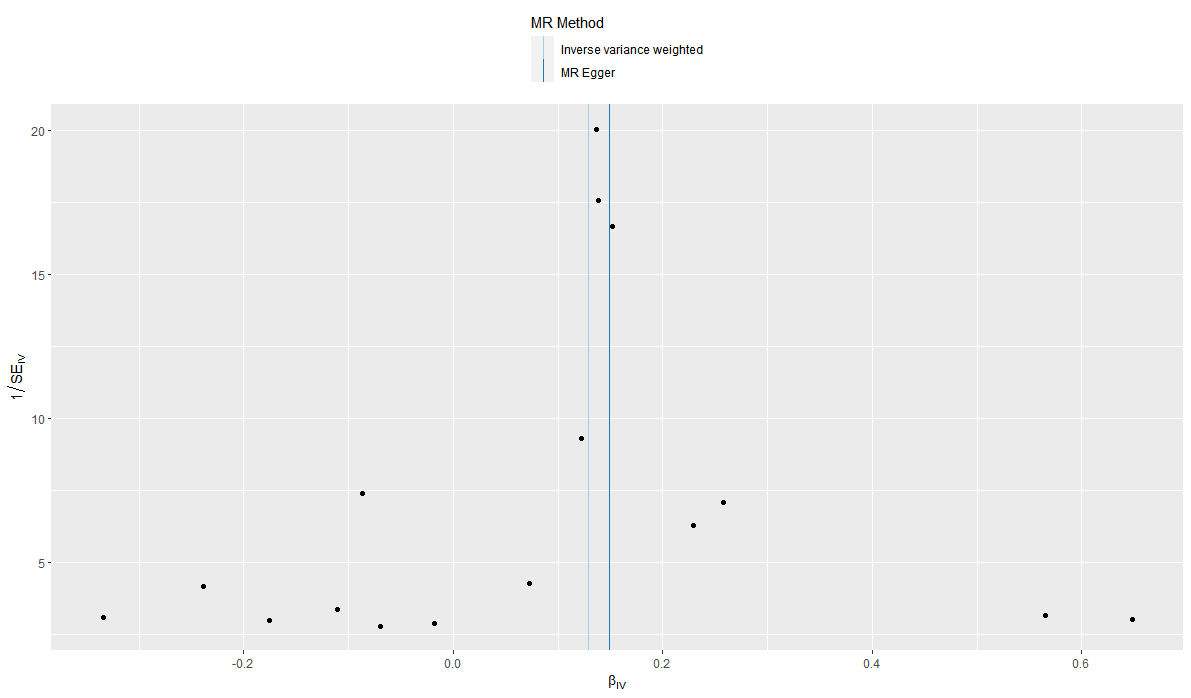


(e) (f) (g) (h)

S.6 Funnel plots of T1DCs from different datasets: (a)Funnel plot of DM1OPTH, (b)Funnel plot of DM1PERIPH, (c)Funnel plot of DM1NASCOMP, (d)Funnel plot of DM1REN, (e)Funnel plot of DMICOMA, (f)Funnel plot of DM1KETO, (g)Funnel plot of DM1NOCOMP, (h)Funnel plot of DM1NEU
